# Varicella encephalitis and pneumonia in a patient with end stage renal failure

**DOI:** 10.1186/1447-056X-13-4

**Published:** 2014-02-21

**Authors:** Lian Leng Low, Farhad Fakhrudin Vasanwala, Sufi Muhammad Suhail

**Affiliations:** 1 Department of Family Medicine and Continuing Care, Singapore General Hospital, Bowyer Block A, Level 2, 169608 Outram Road, Singapore; 2 Department of Renal medicine, Singapore General Hospital, Outram Road, Singapore

**Keywords:** End stage renal failure, Varicella, Vaccination

## Abstract

We describe a patient with end stage renal failure (ESRF) on hemodialysis who was admitted to our department for primary varicella infection complicated by varicella pneumonia and encephalitis. Varicella infections results in serious morbidity and mortality in ESRF dialysis and transplant patients. Evidence published thus far suggests that live attenuated varicella vaccines are effective and safe in ESRF and renal transplant patients. Worldwide a few countries have instituted guidelines for the varicella immunisation in ESRF patients. However, in the Asia Pacific Region, it has not been widely given due to the lack of national consensus guidelines. Our case depicts that primary varicella infection can occur at any time in immunosupressed patients and thus suffer serious consequences from it. With increasing burden of chronic kidney disease, Renal Physicians and Family Physicians in the Asia Pacific Region should meet and study the epidemiological data in each individual country and decide on the consensus guidelines on how the varicella vaccination can be targeted for those at risk.

## Background

Our case report illustrates the serious complications of varicella in a patient with end stage renal failure and emphasizes the need for consensus guidelines on varicella vaccinations in such patients in the Asia Pacific region. Although evidence in literature show that live attenuated vaccines can be safe & effective, these vaccines are still generally avoided due to lack of consensus guidelines on vaccinations in patients with end stage renal failure. Literature review provides evidence on the safety and efficacy of varicella vaccination in end stage renal failure patients. With the increasing burden of chronic kidney disease, family physicians also need to be alert for these complications of varicella and can play a big role in facilitating varicella vaccination in patients with end stage renal failure.

## Case presentation

Our patient is a 58 year old construction supervisor with background of hypertension and ESRF on haemodialysis three times per week. He was admittted via the Accident and Emergency Department to Singapore General Hospital Department of Family Medicine and Continuing Care for fever, chills and non productive cough of two days duration. There was no travel or contact history. On examination, he was alert, non-toxic looking and orientated to time, place and person. He had a temperature of 38.4 degree Celsius, blood pressure of 140/90 mmHg, pulse rate of 80 per minute and respiratory rate of 16 per minute. His respiratory examination revealed reduced chest expansion over the left lung base associated with dullness to percussion, bronchial breath sounds and crackles. His cardiovascular, abdominal and neurological examination was unremarkable. A chest radiograph confirmed consolidation in the left lower zone with milder air space opacity in the right lower zone. He was initially diagnosed with healthcare associated pneumonia in view of recent hospitalization and started on intravenous tazocin. Respiratory viruses multiplex PCR (Polymerase Chain Reaction) was negative.

On the fourth day of admission, he developed vesiculo-papular pruritic lesions mainly over the trunk. Varicella Zoster Virus (VZV) IgM was positive. He was isolated together with air-borne and contact precautions, and started on oral Valacyclovir 500 mg daily later on that day. The next day, his oxygen saturation decreased, and worsening of the consolidation was noted on repeat chest radiograph. He also developed vivid visual hallucinations, became restless and agitated and disorientated to time, place and person. A diagnosis of varicella zoster infection complicated by varicella pneumonia and encephalitis was made. Valacyclovir was switched to intravenous acyclovir. A lumbar puncture showed increased protein and lymphocytes and was negative for cryptococcal antigen and neurotropic viruses. A magnetic resonance imaging scan of the brain showed no evidence of acute infarct, intracranial bleed, space-occupying lesion or hydrocephalus. The frequency of dialysis was increased with continuation of the renal adjusted dose of acyclovir.

Throughout the admission, his hemodialysis continued and he completed 2 weeks of IV acyclovir. His functional status improved back to the pre-morbid levels on discharge.

## Discussion

The incidence of varicella in Singapore has been increasing since 1984 [1] and increased from 14,999 in 2003 to 24,031 in 2006. Mandatory notification of varicella ceased since 27th August 2007 [2]. Thus the latest reported figures are not known.

Primary infection with varicella is usually a benign and self-limiting illness in immunocompetent children. However, patients with end stage renal failure have lymphocytopenia and impaired lymphocyte function and are susceptible for disseminated varicella and its complications, with more severe morbidity and mortality rates [3,4].

Our patient suffered severe complications of varicella infection that is pneumonia and viral encephalitis. Other known complications include myocarditis, corneal lesions, nephritis, arthritis, bleeding diatheses, acute glomerulonephritis, hepatitis and secondary bacterial superinfection of the skin.

The central nervous system is the most common extra cutaneous site of involvement, manifesting as acute cerebellar ataxia or diffuse encephalitis. These disorders typically develop toward the end of the first week of the exanthem, but can also precede the rash [5,6]. Diffuse encephalitis most often occurs in adults and clinical manifestations include delirium, seizures, loss of consciousness and focal neurological signs including cranial nerve palsies, hemiparesis. Varicella encephalitis is more severe in immunosuppressed hosts who typically have a fulminant course with seizures, mental status changes and focal deficits including stroke syndromes. However very little data exist regarding the occurrence of varicella encephalitis in patients with end stage renal failure or those undergoing dialysis.

In the workup for VZV encephalitis, computed tomography or magnetic resonance imaging may or may not demonstrate abnormal radiographic findings, although electroencephalography is often abnormal in acute encephalitis. The cerebrospinal fluid contains lymphocytes and elevated levels of protein but normal glucose concentration, allowing differentiation from bacterial meningitis. Cerebrospinal fluid polymerase chain reaction (CSF PCR) can be used to detect VZV DNA, and has a specificity of greater than 95%, but the sensitivity is 30% or less in some studies [7]. Repeated mortality rates approach 10% and long-term neurologic sequelae are reported in up to 15% of survivors [8-10]. There is no proven effective therapy once encephalitis occurs, and supportive care remains the mainstay of management. Acyclovir has been used with anecdotal success [11-13]. However, acyclovir neurotoxicity should always be considered in patients with prolonged or worsening neurological symptoms.

Varicella pneumonia is the most serious complication following varicella, develops more commonly in adults (up to 20% of cases) than in children, and accounts for the majority of morbidity and mortality seen in adults with varicella [11]. Risk factors linked to the development of varicella pneumonia include cigarette smoking, pregnancy and an immunosuppressed state [9]. Varicella pneumonia typically develops insidiously with symptoms of progressive dyspnea and dry cough. Patients demonstrate impaired gas exchange with progressive hypoxemia and are at high risk for respiratory failure and need for admission to intensive care for mechanical ventilation [14,15]. Mortality rates approach 50% in patients with respiratory failure who require mechanical ventilation, despite institution of aggressive therapy and appropriate support measures [16,17]. Chest radiographs typically reveal diffuse bilateral infiltrates. Prompt administration of intravenous acyclovir has been associated with clinical improvement and resolution of pneumonia in selected series [17,18]. Resolution of pneumonitis parallels improvement of the skin rash, although fever and compromised pulmonary function may persist for weeks [11]. The use of steroids as adjunctive therapy for treatment of life-threatening varicella pneumonia is still controversial and should be further examined in rigorous controlled trials [19].

In immunosuppressed hosts such as ESRF patients or immunocompetent patients with disseminated disease such as pneumonia or encephalitis, varicella should be treated with IV acyclovir [20]. IV acyclovir reduces the occurrence of visceral complications but has no effect on healing of skin lesions or pain [11]. The recommended dose is 10 mg/kg Q8h for 7 days.

In December 2012, the FDA approved varicella zoster immune globulin (VariZIG) for administration to high risk individuals within 4 days of varicella exposure.

Acyclovir is an effective agent for the treatment of VZV infections but renal impairment results in high serum drug levels with resultant neurotoxicity. In renal failure, the half-life of acyclovir is increased from a maximum of 3.8 to 20 hours, and dosage reduction is required using the interval extension method [21]. Even the administration of recommended reduced doses can result in high serum levels and neurotoxicity, due to poor removal of acyclovir by peritoneal dialysis [4]. Tremors, disorientation, agitation, hallucinations, and delirium are common presentations of acyclovir-induced encephalopathy, whereas seizures, cerebellar ataxia, sensory symptoms, speech disorders, fever and cranial nerve palsies are much less frequent. Features distinguishing acyclovir neurotoxicity from VZV encephalitis include a temporal association between the symptoms and acyclovir use, as well as acellular CSF examination. However, acyclovir neurotoxicity should always be considered in patients with prolonged or worsening neurological symptoms for which daily hemodialysis is required often.

Studies show that many ESRF and kidney transplant patients do not have immunity to varicella [22-25]. Primary varicella infection is common, up to 1.9% of renal transplant recipients are admitted in the first year post-transplant [26]. The infection can have serious complications and be lethal in these ESRF and renal transplant patients who are immunosuppressed [24,26-29].

Live attenuated varicella vaccine is generally contraindicated in immunosuppressed patients but has been proven to be safe when administered to both pediatric [23,24,29] and adult end stage renal failure patients on dialysis, [27] as well as renal transplant patients on immunosuppressive treatment [25].

Despite the general suppression of the immune system associated with uremia, patients with ESRF have high seroconversion rates to a two-dose varicella vaccination regimen. Seroconversion rates ranged from 87-100% [23,29-31] in ESRF pediatric patients waiting for transplant and 64% in adult ESRF patients [24]. 66.6% of pediatric renal transplant recipients seroconverted after a one–two dose varicella vaccination regimen [25]. Immunization guidelines in New Zealand recommend a two dose varicella vaccination regimen for children with deteriorating renal function, as early as possible before transplantation [32]. In the United States, the Centers for Disease Control and Prevention (CDC) adult immunization schedule recommend a two dose varicella vaccination regimen and a single dose zoster vaccination for patients with ESRF and recipients of hemodialysis [33,34]. However, ESRF patients have an inability to maintain adequate antibody titers over time [35,36]. Reports describe several patients in whom immunity to varicella waned in chronic renal insufficiency and in the post-transplant period [26,27]. Hence there is a need for yearly surveillance of antibody titre as are done for Hepatitis B & C virus and cytomegalovirus. Special consideration for vaccination should also be given to persons who have close contact with or are at high risk of transmission to ESRF patients [32]. Although there are recommendations to administer varicella immunoglobulin to transplant patient coming in contact with varicella patients [37], the use of immunglobulins has risks and is expensive. Immunoglobulins should be reserved for those with imminent exposure to the virus. Varicella vaccination is a cheaper and more effective way for prevention of varicella.

### Role of family physicians

There were 4,169 patients on dialysis in Singapore by 2008. This number represented a 89% increase since end 1998, when there were 2,209 patients on dialysis. The incidence of new ESRF patients requiring dialysis increases every year, from 564 new cases in 1998 to 1,212 cases in 2008. This represents a 115% increase during this 10-year period [38-40]. In addition, patients are getting more elderly. In 2006, 52.1% and 5.1% of incident dialysis patients were over the age of 60 and 80 respectively. With a rapidly ageing population, Singapore can expect an increasing burden of end stage renal disease Family physicians should advise their ESRF patients of the life-threatening complications of varicella and urgent need for review if they develop fever or rash after coming in contact with varicella patients.

Currently, there are no local data on the seropositivity against varicella antibody in ESRF patients in Singapore or consensus guidelines for varicella vaccination. With an increasing burden of chronic kidney disease, Renal Physicians and Family Physicians in the Asia-Pacific region should meet and study the epidemiological data in each individual country and decide on consensus guidelines and how the varicella vaccination program can be targeted for those at risk. Research on the seroprevalence of varicella in ESRF patients should be conducted if such data is not currently available in Asia-Pacific countries.

## Conclusions

Evidence supports the safety and efficacy of varicella vaccination in patients with end stage renal failure. Guidelines for varicella vaccination for ESRF patients are available in a few countries [33,34]. The intent of our paper is to stimulate more research in the area of generating the need of consensus guidelines for universal vaccinations of ESRF patients on dialysis. Serious consequences of primary varicella infection can be avoided with appropriate vaccinations.

### Consent

Written informed consent was obtained from the patient for publication of this case report and any accompanying images. A copy of the written consent is available for review by the Editor-in-Chief of this journal.

## Abbreviations

ESRF: End stage renal failure; VZV: Varicella-Zoster virus.

## Competing interests

The authors declare that they have no competing interests.

## Authors’ contributions

LLL, FFV and SMS participated in the proposal, design, and drafted the manuscript. All authors read and approved of the final manuscript.
